# 4-Amino-3-ammonio­benzene­sulfonate

**DOI:** 10.1107/S1600536810048063

**Published:** 2010-11-24

**Authors:** Jian-Lian Liu, Chao-Jun Du, Li-Sheng Wang

**Affiliations:** a Department of Chemical and Biochemical Engineering, Nanyang Institute of Technology, 473004 Nanyang, Henan, People’s Republic of China; b School of Chemical Engineering and Environment, Beijing Institute of Technology, 100081 Beijing, People’s Republic of China

## Abstract

The title compound, C_6_H_8_N_2_O_3_S, crystallized as a sulfonate–aminium zwitterion. In the crystal, inter­molecular N—H⋯O hydrogen bonds generate an extensive three-dimensional network, which consolidates the packing.

## Related literature

For the crystal structures of isomers of the title compound, see: Rubin-Preminger & Bernstein (2003 ▶). For details of the synthesis, see: Miranda *et al.* (2008 ▶).
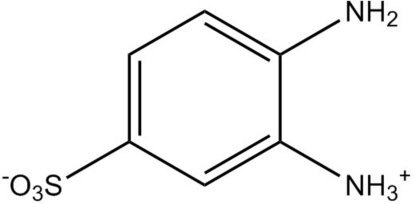


## Experimental

### 

#### Crystal data

C_6_H_8_N_2_O_3_S
*M* *_r_* = 188.20
Monoclinic, 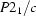

*a* = 5.602 (1) Å
*b* = 8.4135 (15) Å
*c* = 16.221 (3) Å
β = 95.613 (2)°
*V* = 760.9 (2) Å^3^
*Z* = 4
Mo *K*α radiation
μ = 0.39 mm^−1^
*T* = 295 K
0.35 × 0.25 × 0.15 mm

#### Data collection

Bruker SMART APEXII CCD area-detector diffractometer
Absorption correction: multi-scan (*SADABS*; Bruker, 2005 ▶) *T*_min_ = 0.875, *T*_max_ = 0.944
4039 measured reflections
1490 independent reflections
1351 reflections with *I* > 2σ(*I*)
*R*_int_ = 0.019

#### Refinement

*R*[*F* ^2^ > 2σ(*F* ^2^)] = 0.031
*wR*(*F* ^2^) = 0.087
*S* = 1.09
1490 reflections
122 parameters
H atoms treated by a mixture of independent and constrained refinement
Δρ_max_ = 0.34 e Å^−3^
Δρ_min_ = −0.33 e Å^−3^

### 

Data collection: *APEX2* (Bruker, 2005 ▶); cell refinement: *SAINT* (Bruker, 2005 ▶); data reduction: *SAINT*; program(s) used to solve structure: *SHELXTL* (Sheldrick, 2008 ▶); program(s) used to refine structure: *SHELXTL*; molecular graphics: *SHELXTL*; software used to prepare material for publication: *SHELXTL*.

## Supplementary Material

Crystal structure: contains datablocks global, I. DOI: 10.1107/S1600536810048063/cv2799sup1.cif

Structure factors: contains datablocks I. DOI: 10.1107/S1600536810048063/cv2799Isup2.hkl

Additional supplementary materials:  crystallographic information; 3D view; checkCIF report

## Figures and Tables

**Table 1:** Hydrogen-bond geometry (Å, °)

*D*—H⋯*A*	*D*—H	H⋯*A*	*D*⋯*A*	*D*—H⋯*A*
N1—H1*B*⋯O1^i^	0.92 (3)	1.86 (3)	2.778 (2)	178 (2)
N1—H1*C*⋯O1^ii^	0.92 (3)	1.89 (3)	2.792 (2)	165 (2)
N1—H1*A*⋯O2^iii^	0.90 (2)	1.88 (3)	2.759 (2)	165 (2)
N2—H2*B*⋯O3^iv^	0.86	2.46	3.047 (2)	126

Symmetry codes: (i) 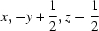; (ii) 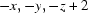; (iii) 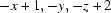; (iv) 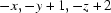.

**Fig. 1.**
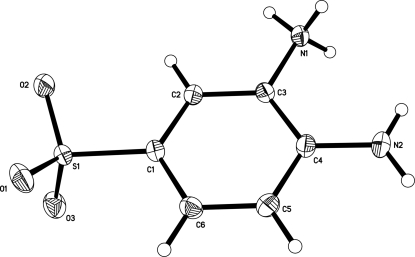
The molecular structure of (I), with displacement ellipsoids drawn at the 30% probability level.

## supplementary crystallographic information

### Comment

The title compound (I) (Fig. 1) is a zwitterion of 4-amino-3-ammoniobenzenesulfonate. The bond lengths and angles in (I) are normal and comparable with those observed in the related compounds (Rubin-Preminger & Bernstein, 2003). In the crystal structure, intermolecular N—H···O hydrogen bonds generate an extensive three-dumensional network which consolidate the crystal packing.

### Experimental

The title compound was synthesized according to the method reported in the literature (Miranda *et al.*, 2008). Orange single crystals suitable for X-ray diffraction were obtained by slow evaporation of a water solution of the compound.

### Refinement

C-bound H atoms and *N*(amino)-bound H atoms were geometrically positioned (C—H = 0.93 Å, N—H = 0.86 Å) and included in the riding model approximation, with *U*_iso_(H) = 1.2 *U*_eq_(C, N). H atoms attached to *N*(ammonio) were located from an electron density map, and isotropically refined with the N—H bond length restrained to 0.91 (3) Å.

### Crystal data

**Table:**

C_6_H_8_N_2_O_3_S	*F*(000) = 392
*M**_r_* = 188.20	*D*_x_ = 1.643 Mg m^−^^3^
Monoclinic, *P*2_1_/*c*	Mo *K*α radiation, λ = 0.71073 Å
Hall symbol: -P 2ybc	Cell parameters from 2332 reflections
*a* = 5.602 (1) Å	θ = 2.4–27.7°
*b* = 8.4135 (15) Å	µ = 0.39 mm^−^^1^
*c* = 16.221 (3) Å	*T* = 295 K
β = 95.613 (2)°	Block, orange
*V* = 760.9 (2) Å^3^	0.35 × 0.25 × 0.15 mm
*Z* = 4	

### Data collection

**Table:**

Bruker SMART APEXII CCD area-detector diffractometer	1490 independent reflections
Radiation source: fine-focus sealed tube	1351 reflections with *I* > 2σ(*I*)
graphite	*R*_int_ = 0.019
phi and ω scans	θ_max_ = 26.0°, θ_min_ = 2.5°
Absorption correction: multi-scan (*SADABS*; Bruker, 2005)	*h* = −6→6
*T*_min_ = 0.875, *T*_max_ = 0.944	*k* = −9→10
4039 measured reflections	*l* = −17→19

### Refinement

**Table:**

Refinement on *F*^2^	Secondary atom site location: difference Fourier map
Least-squares matrix: full	Hydrogen site location: inferred from neighbouring sites
*R*[*F*^2^ > 2σ(*F*^2^)] = 0.031	H atoms treated by a mixture of independent and constrained refinement
*wR*(*F*^2^) = 0.087	*w* = 1/[σ^2^(*F*_o_^2^) + (0.0446*P*)^2^ + 0.3348*P*] where *P* = (*F*_o_^2^ + 2*F*_c_^2^)/3
*S* = 1.09	(Δ/σ)_max_ = 0.001
1490 reflections	Δρ_max_ = 0.34 e Å^−^^3^
122 parameters	Δρ_min_ = −0.33 e Å^−^^3^
0 restraints	Extinction correction: *SHELXTL* (Sheldrick, 2008), Fc^*^=kFc[1+0.001xFc^2^λ^3^/sin(2θ)]^-1/4^
Primary atom site location: structure-invariant direct methods	Extinction coefficient: 0.022 (3)

### Special details

**Table:**

Geometry. All e.s.d.'s (except the e.s.d. in the dihedral angle between two l.s. planes) are estimated using the full covariance matrix. The cell e.s.d.'s are taken into account individually in the estimation of e.s.d.'s in distances, angles and torsion angles; correlations between e.s.d.'s in cell parameters are only used when they are defined by crystal symmetry. An approximate (isotropic) treatment of cell e.s.d.'s is used for estimating e.s.d.'s involving l.s. planes.
Refinement. Refinement of *F*^2^ against ALL reflections. The weighted *R*-factor *wR* and goodness of fit *S* are based on *F*^2^, conventional *R*-factors *R* are based on *F*, with *F* set to zero for negative *F*^2^. The threshold expression of *F*^2^ > σ(*F*^2^) is used only for calculating *R*-factors(gt) *etc*. and is not relevant to the choice of reflections for refinement. *R*-factors based on *F*^2^ are statistically about twice as large as those based on *F*, and *R*- factors based on ALL data will be even larger.

### Fractional atomic coordinates and isotropic or equivalent isotropic displacement parameters (Å^2^)

**Table:**

	*x*	*y*	*z*	*U*_iso_*/*U*_eq_	
C1	0.1329 (3)	0.2272 (2)	1.04906 (10)	0.0265 (4)	
C2	0.1963 (3)	0.15996 (19)	0.97641 (10)	0.0263 (4)	
H2	0.3348	0.0988	0.9769	0.032*	
C3	0.0532 (3)	0.18407 (19)	0.90339 (10)	0.0263 (4)	
C4	−0.1567 (3)	0.2754 (2)	0.90017 (10)	0.0300 (4)	
C5	−0.2178 (3)	0.3403 (2)	0.97466 (11)	0.0341 (4)	
H5	−0.3570	0.4005	0.9747	0.041*	
C6	−0.0764 (3)	0.3168 (2)	1.04811 (11)	0.0314 (4)	
H6	−0.1207	0.3608	1.0970	0.038*	
H1A	0.266 (4)	0.065 (3)	0.8377 (14)	0.052 (7)*	
H1B	0.132 (4)	0.185 (3)	0.7858 (16)	0.045 (6)*	
H1C	0.012 (4)	0.033 (3)	0.8111 (15)	0.055 (7)*	
N1	0.1219 (3)	0.1112 (2)	0.82725 (9)	0.0304 (3)	
N2	−0.2985 (3)	0.3022 (2)	0.82732 (11)	0.0502 (5)	
H2B	−0.4260	0.3592	0.8274	0.060*	
H2A	−0.2597	0.2619	0.7818	0.060*	
O1	0.1572 (3)	0.16078 (16)	1.20518 (8)	0.0413 (4)	
O2	0.4779 (3)	0.07161 (18)	1.12735 (8)	0.0518 (4)	
O3	0.4413 (2)	0.34942 (17)	1.16250 (8)	0.0424 (4)	
S1	0.31916 (8)	0.20155 (5)	1.14202 (2)	0.02874 (18)	

### Atomic displacement parameters (Å^2^)

**Table:**

	*U*^11^	*U*^22^	*U*^33^	*U*^12^	*U*^13^	*U*^23^
C1	0.0281 (9)	0.0290 (8)	0.0218 (8)	0.0004 (6)	−0.0011 (6)	−0.0005 (6)
C2	0.0269 (8)	0.0274 (8)	0.0243 (8)	0.0028 (6)	0.0004 (6)	0.0009 (6)
C3	0.0302 (9)	0.0259 (8)	0.0222 (8)	0.0004 (6)	0.0005 (6)	−0.0006 (6)
C4	0.0303 (9)	0.0298 (8)	0.0285 (9)	0.0012 (7)	−0.0045 (7)	0.0012 (7)
C5	0.0277 (9)	0.0369 (9)	0.0368 (10)	0.0073 (7)	−0.0004 (7)	−0.0027 (7)
C6	0.0309 (9)	0.0363 (9)	0.0275 (9)	0.0036 (7)	0.0045 (7)	−0.0054 (7)
N1	0.0352 (9)	0.0334 (8)	0.0217 (7)	0.0054 (7)	−0.0010 (6)	−0.0001 (6)
N2	0.0538 (11)	0.0559 (11)	0.0364 (9)	0.0241 (9)	−0.0188 (8)	−0.0073 (8)
O1	0.0556 (9)	0.0457 (8)	0.0228 (6)	−0.0086 (6)	0.0054 (6)	−0.0029 (5)
O2	0.0625 (9)	0.0574 (9)	0.0324 (7)	0.0326 (8)	−0.0104 (6)	−0.0080 (6)
O3	0.0452 (8)	0.0432 (8)	0.0366 (7)	−0.0086 (6)	−0.0072 (6)	−0.0032 (6)
S1	0.0343 (3)	0.0307 (3)	0.0203 (2)	0.00447 (16)	−0.00195 (17)	−0.00273 (15)

### Geometric parameters (Å, °)

**Table:**

C1—C2	1.385 (2)	C5—H5	0.9300
C1—C6	1.393 (2)	C6—H6	0.9300
C1—S1	1.7611 (17)	N1—H1A	0.90 (2)
C2—C3	1.379 (2)	N1—H1B	0.92 (3)
C2—H2	0.9300	N1—H1C	0.92 (3)
C3—C4	1.401 (2)	N2—H2B	0.8600
C3—N1	1.464 (2)	N2—H2A	0.8600
C4—N2	1.376 (2)	O1—S1	1.4739 (14)
C4—C5	1.399 (2)	O2—S1	1.4435 (14)
C5—C6	1.379 (2)	O3—S1	1.4428 (14)
			
C2—C1—C6	119.85 (15)	C1—C6—H6	120.0
C2—C1—S1	119.92 (13)	C3—N1—H1A	109.1 (15)
C6—C1—S1	120.23 (13)	C3—N1—H1B	111.8 (14)
C3—C2—C1	119.66 (15)	H1A—N1—H1B	108 (2)
C3—C2—H2	120.2	C3—N1—H1C	108.5 (14)
C1—C2—H2	120.2	H1A—N1—H1C	108 (2)
C2—C3—C4	121.88 (15)	H1B—N1—H1C	111 (2)
C2—C3—N1	118.96 (15)	C4—N2—H2B	120.0
C4—C3—N1	119.16 (15)	C4—N2—H2A	120.0
N2—C4—C5	120.55 (16)	H2B—N2—H2A	120.0
N2—C4—C3	122.25 (16)	O3—S1—O2	113.91 (10)
C5—C4—C3	117.20 (15)	O3—S1—O1	110.55 (8)
C6—C5—C4	121.48 (16)	O2—S1—O1	111.67 (9)
C6—C5—H5	119.3	O3—S1—C1	108.61 (8)
C4—C5—H5	119.3	O2—S1—C1	105.94 (8)
C5—C6—C1	119.93 (16)	O1—S1—C1	105.68 (8)
C5—C6—H6	120.0		
			
C6—C1—C2—C3	0.9 (3)	C4—C5—C6—C1	0.2 (3)
S1—C1—C2—C3	−178.25 (13)	C2—C1—C6—C5	−1.0 (3)
C1—C2—C3—C4	0.0 (3)	S1—C1—C6—C5	178.15 (14)
C1—C2—C3—N1	−179.58 (15)	C2—C1—S1—O3	106.55 (15)
C2—C3—C4—N2	178.93 (17)	C6—C1—S1—O3	−72.59 (16)
N1—C3—C4—N2	−1.5 (3)	C2—C1—S1—O2	−16.19 (17)
C2—C3—C4—C5	−0.8 (3)	C6—C1—S1—O2	164.66 (15)
N1—C3—C4—C5	178.80 (16)	C2—C1—S1—O1	−134.81 (14)
N2—C4—C5—C6	−179.04 (18)	C6—C1—S1—O1	46.04 (17)
C3—C4—C5—C6	0.7 (3)		

### Hydrogen-bond geometry (Å, °)

**Table:**

*D*—H···*A*	*D*—H	H···*A*	*D*···*A*	*D*—H···*A*
N1—H1B···O1^i^	0.92 (3)	1.86 (3)	2.778 (2)	178 (2)
N1—H1C···O1^ii^	0.92 (3)	1.89 (3)	2.792 (2)	165 (2)
N1—H1A···O2^iii^	0.90 (2)	1.88 (3)	2.759 (2)	165 (2)
N2—H2B···O3^iv^	0.86	2.46	3.047 (2)	126

Symmetry codes: (i) *x*, −*y*+1/2, *z*−1/2; (ii) −*x*, −*y*, −*z*+2; (iii) −*x*+1, −*y*, −*z*+2; (iv) −*x*, −*y*+1, −*z*+2.
